# Effectiveness of radiation protection systems in the cardiac catheterization laboratory: a comparative study

**DOI:** 10.1007/s00392-022-02142-8

**Published:** 2023-01-16

**Authors:** Victoria L. Cammann, Victor Schweiger, Maciej Cieslik, Burkhardt Seifert, Thomas Gilhofer, Iva Koleva, Michael Würdinger, Alessandro Candreva, Marko Gajic, Jonathan Michel, Philipp Jakob, Julia Stehli, Barbara Stähli, Christian Templin, Alexander Gotschy

**Affiliations:** 1 Department of Cardiology, University Hospital Zurich, University Heart Center, Raemistrasse 100, 8091 Zurich, Switzerland; 2 University of Zurich, Zurich, Switzerland; 3 Division of Biostatistics, Epidemiology, Biostatistics and Prevention Institute, University of Zurich, Zurich, Switzerland; 4 PolitoBIO Med Lab, Department of Mechanical and Aerospace Engineering, Politecnico di Torino, Turin, Italy; 5 Institute of Diagnostic and Interventional Radiology, University Hospital Zurich, Zurich, Switzerland; 6 Institute for Biomedical Engineering, University and ETH Zurich, Zurich, Switzerland

**Keywords:** Radiation protection, Suspended radiation protection systems, SRPS, Protective scatter-radiation absorbing drapes, RADPAD, Cardiac catheterization

## Abstract

**Background:**

As numbers and complexity of percutaneous coronary interventions are constantly increasing, optimal radiation protection is required to ensure operator safety. Suspended radiation protection systems (SRPS) and protective scatter-radiation absorbing drapes (PAD) are novel methods to mitigate fluoroscopic scattered radiation exposure. The aim of the study was to investigate the effectiveness regarding radiation protection of a SRPS and a PAD in comparison with conventional protection.

**Methods:**

A total of 229 cardiac catheterization procedures with SRPS (*N* = 73), PAD (*N* = 82) and standard radiation protection (*N* = 74) were prospectively included. Real-time dosimeter data were collected from the first operator and the assistant. Endpoints were the cumulative operator exposure relative to the dose area product [standardized operator exposure (SOE)] for the first operator and the assistant.

**Results:**

For the first operator, the SRPS and the PAD significantly decreased the overall SOE compared to conventional shielding by 93.9% and 66.4%, respectively (*P* < 0.001). The protective effect of the SRPS was significantly higher compared to the PAD (*P* < 0.001). For the assistant, the SRPS and the PAD provided a not statistically significant reduction compared to conventional shielding in the overall SOE by 38.0% and 30.6%, respectively.

**Conclusions:**

The SRPS and the PAD enhance radiation protection significantly compared to conventional protection. In most clinical scenarios, the protective effect of SRPS is significantly higher than the additional protection provided by the PAD.

**Graphical abstract:**

**Figure:**
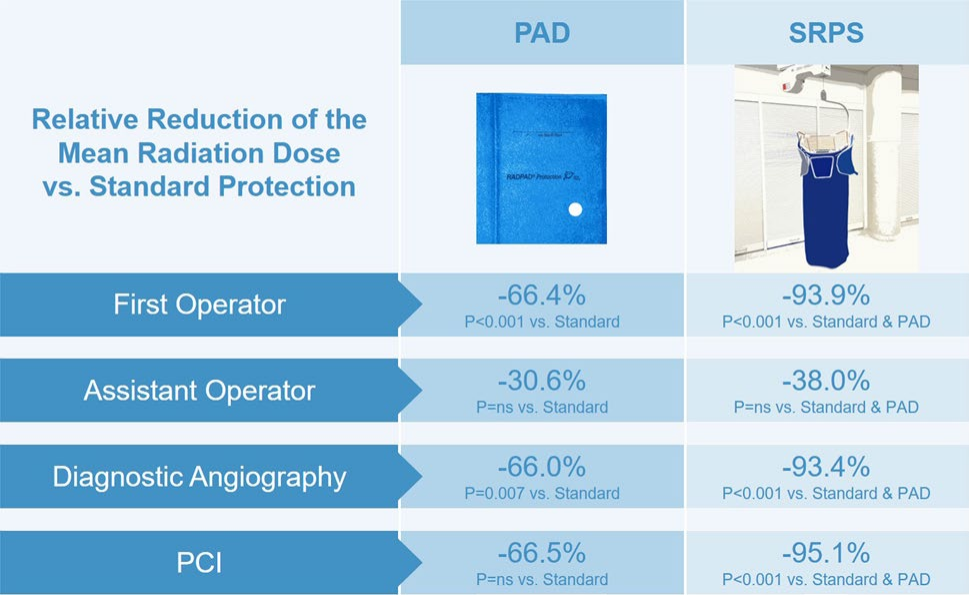
Comparison of the additional radiation protection provided by protective scatter-radiation absorbing drapes (PAD) and the suspended radiation protection system (SRPS) system over standard protection with lead aprons.

**Supplementary Information:**

The online version contains supplementary material available at 10.1007/s00392-022-02142-8.

## Introduction

Cardiac catheterization procedures can cause harm to the physicians performing the procedures as they are exposed to significant doses of scattered radiation. The amount of received radiation depends on the fluoroscopy system used, the type of procedure, and patients’ characteristics [1, 2]. While technical advances led to an overall reduction of percutaneous coronary intervention (PCI) related radiation exposure over the last decade, the received dose in complex interventions such as chronic total occlusion (CTO)-PCI or multivessel PCI remains high [3, 4]. Considering the constantly growing number and complexity of cardiac catheterization procedures performed in a population with a rising prevalence of obesity, the importance of adequate protection from fluoroscopic scatter radiation remains of utmost importance. Scattered radiation may cause a wide range of occupational health hazards including premature cataract formation, subclinical atherosclerosis, or an increased risk of malignancies in interventionalists [5–8].

In order to protect interventionalists from scatter radiation, several protective devices have been developed, such as protective scatter radiation-absorbing drapes (PAD) and suspended radiation protection systems (SRPS). The PAD is a sterile, lead-free, disposable drape that is placed on the patient at the level of the puncture site. The SRPS is a suspension system carrying a movable 1-mm-thick lead body shield that provides strong protection from fluoroscopic scatter-radiation while reducing the weight on the operator and allowing a high degree of freedom of movement. Previous studies on interventional radiological procedures demonstrated that scattered radiation reaching the eye can be significantly reduced by the use of the SRPS, while the use of the PAD has also been associated with reduced operator dose in cardiac catheterization laboratories [9, 10]. Currently, no direct comparison of the SRPS and PAD to conventional protection measure has been performed. Therefore, the present study aimed to investigate the protection from fluoroscopic scatter radiation provided by the SRPS and PAD in comparison with conventional shielding during coronary angiography.

## Methods

### Study design

The present study was prospectively performed at the Andreas Grüntzig cardiac catheterization laboratories at the University Hospital Zurich, Switzerland. Emergency and elective diagnostic coronary angiographies (CAG) and PCI were included in the study. Procedures were assigned in a 1:1:1 ratio to the SRPS, PAD and a control group for which conventional radiation protection measures were used. Patients were assigned to their respective group in consecutive blocks, aiming for 80 patients per group. It was left at the discretion of the operator not to included patients for clinical reasons (e.g. hemodynamic instability, ongoing resuscitation, …). The operators were free to choose the access site according to clinical considerations and operator experience. Exclusion criteria were (1) change in the first operator during the procedure or (2) crossover from radial/brachial to femoral access. Upon request to the cantonal ethics committee Zurich, ethical approval was waived as the study does not fall within the scope of the Human Research Act.

### Study setting

**Fig. 1:**
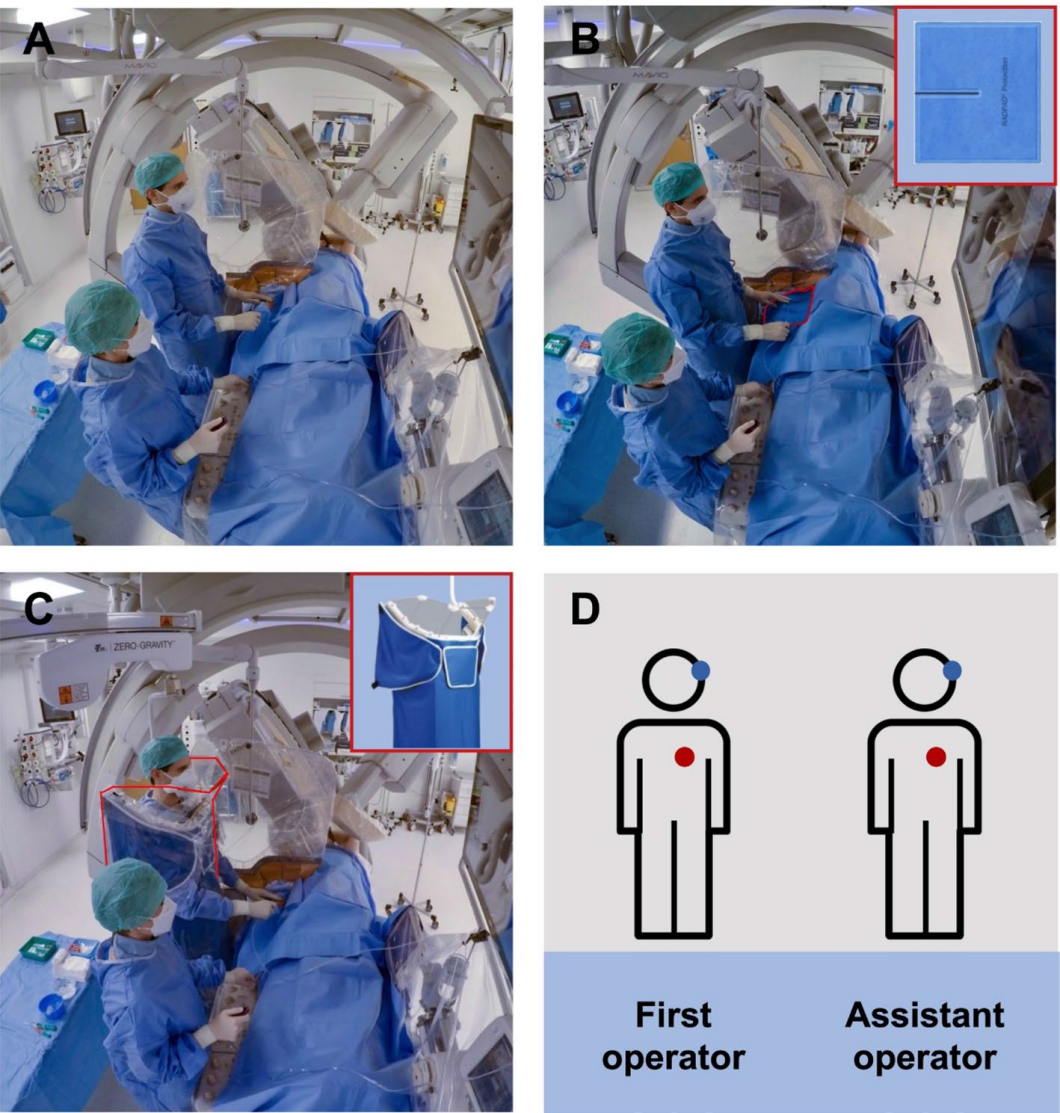
Study devices and dosimeter placement. Representative situation of a control intervention with conventional protection (**A**), positioning of the PAD (Multipurpose Shield, Worldwide Innovations & Technologies, Inc., USA) (**B**) and alignment of the SRPS (BIOTRONIK SE & Co. KG, Germany) (**C**). **D** Depicts the positioning of the dosimeters of the first operator and the assistant (red dots: indicate placement underneath the lead apron; blue dots: indicate placement above protective gear). If the SRPS was used the first operator wore the chest dosimeter underneath the SRPS. *PAD* protective scatter-radiation absorbing drapes, *SRPS* suspended radiation protection system

All procedures were performed in the two catheterization rooms with identical Philips Allura Xper FD10 bi-plane X-ray systems (Philips Medical Systems, Switzerland). The systems are equipped with three auxiliary shields, one under the table, one on the side of the table, and a mobile suspended acrylic shield. The specific radiation protection measures for each group were as follows:

Control group: The first operator wore a conventional lead apron and a thyroid shield. The moveable radiation protection shields were positioned optimally for each patient (Fig. 1A).

PAD group: In addition to the measures of the control group, a disposable protective shield (RADPAD^®^ Femoral Entry Shield or RADPAD^®^ Multipurpose Shield, Worldwide Innovations & Technologies, Inc., USA) was positioned on the patient around the sheath insertion point and just caudal to the area covered by the suspended acrylic shield. Optimal positioning of the PAD was repeatedly checked during the procedure (Fig. 1B).

SRPS group: The first operator did not wear a lead apron or a thyroid shield. Instead, a light vest (Supplementary Fig. 1), which was magnetically connected to the Zero-Gravity (BIOTRONIK SE & Co. KG, Germany) system was worn. The height of the system was adjusted for each operator to ensure optimal protection. In addition, the moveable radiation protection shields were positioned as in both other groups (Fig. 1C).

The assistant wore a conventional lead apron and a thyroid shield in all three study groups. All aprons had an equivalence of 0.5 mm Pb. Exposure data were collected using electronic real-time dosimeters (RaySafe i3, Unfors RaySafe AB, Sweden). The first operator and the assistant wore two dosimeter one at eye-level at the left side of the head and one at the chest level under the lead apron or under the SRPS, respectively (Fig. 1D).

### Study endpoints and definitions

The primary endpoint was the standardized operator exposure (SOE, sum of head and chest exposure) of the first operator and the secondary endpoint was the SOE received by the assistant. In subgroup analyses, differences in the SOE of the first operator between the SRPS, PAD, and control group were analyzed in various procedural settings (emergency and elective CAG; diagnostic CAG and PCI; radial/brachial access and femoral access). As in other studies on occupational radiation exposure during interventional procedures, the exposure was normalized to the dose area product (DAP) as this most accurately reflects operator radiation exposure independent of fluoroscopy time and other complicating factors [9, 11, 12]. The SOE indicates the cumulative operator exposure relative to the DAP, which is calculated as the absorbed skin entrance dose multiplied by the irradiated area and therefore expressed µSv/Gy cm^2^ (i.e. the amount of radiation received per Gy cm^2^ DAP).

The first operator was defined as the interventional cardiologist who performed the procedure and was standing closest to the X-ray system for the entire procedure. The assistant was defined as the person standing on the right side of the first operator and remaining in proximity to the catheterization table throughout the procedure. Patients’ and procedural characteristics such as fluoroscopy time, DAP, body mass index (BMI), and cardiovascular risk factors were collected and analyzed between the three study groups.

### Statistical analysis

**Fig. 2:**
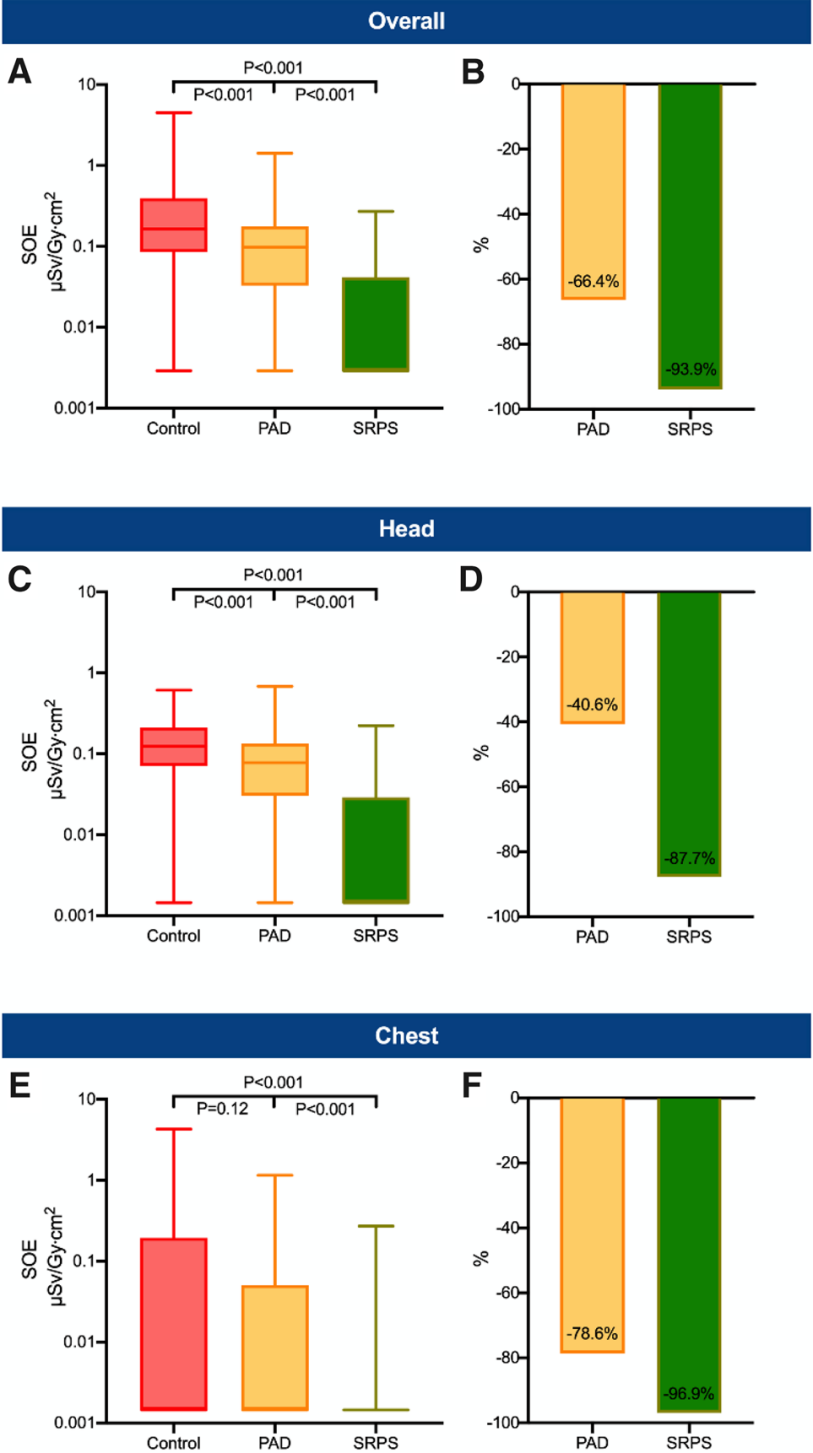
Standardized operator exposure of the first operator. Overall (head and chest) standardized operator exposure (SOE) (**A**) as well as SOE at head (**C**) and chest level (**E**) of the first operator. The lower whisker shows the minimum and the upper whisker the maximum. Boxes extend from the 25th to the 75th percentile. Horizontal line in the box shows the median (50th percentile). Values with SOE = 0 µSv/Gy cm^2^ were replaced with half of the minimum detectable SOE value for illustration on the log-scale. Bar charts show the overall relative reduction in the mean SOE (**B**) as well as the relative reduction in the mean SOE at the head (**D**) and chest level (**F**). Please note that the median SOE of the chest dosimeters in all groups and the head dosimeter in the SRPS group was 0 µSv/Gy cm^2^ and was set to half of the detection limit of the dosimeters accordingly. *PAD* protective scatter-radiation absorbing drapes, *SRPS* suspended radiation protection system

**Fig. 3:**
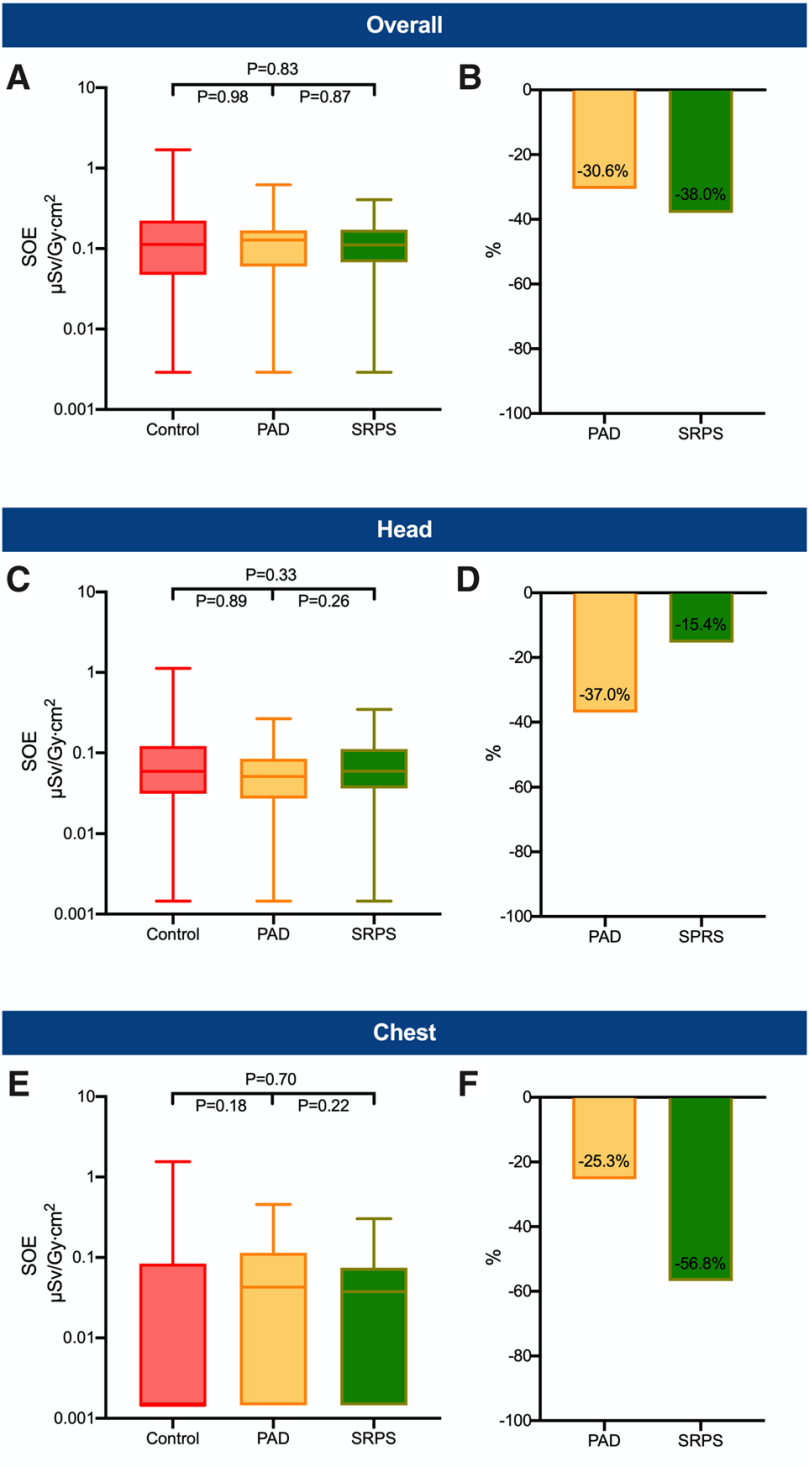
Standardized operator exposure of the assistant. Overall (head and chest) standardized operator exposure (SOE) (**A**) as well as SOE at head (**C**) and chest level (**E**) of the assistant. The lower whisker shows the minimum and the upper whisker the maximum. Boxes extend from the 25th to the 75th percentile. Horizontal line in the box shows the median (50th percentile). Values with SOE = 0 µSv/Gy cm^2^ were replaced with half of the minimum detectable SOE value for illustration on the log-scale. Bar charts show the overall relative reduction in the mean SOE (**B**) as well as the relative reduction in the mean SOE at the head (**D**) and chest level (**F**). *PAD* protective scatter-radiation absorbing drapes, *SRPS* suspended radiation protection system

**Fig. 4:**
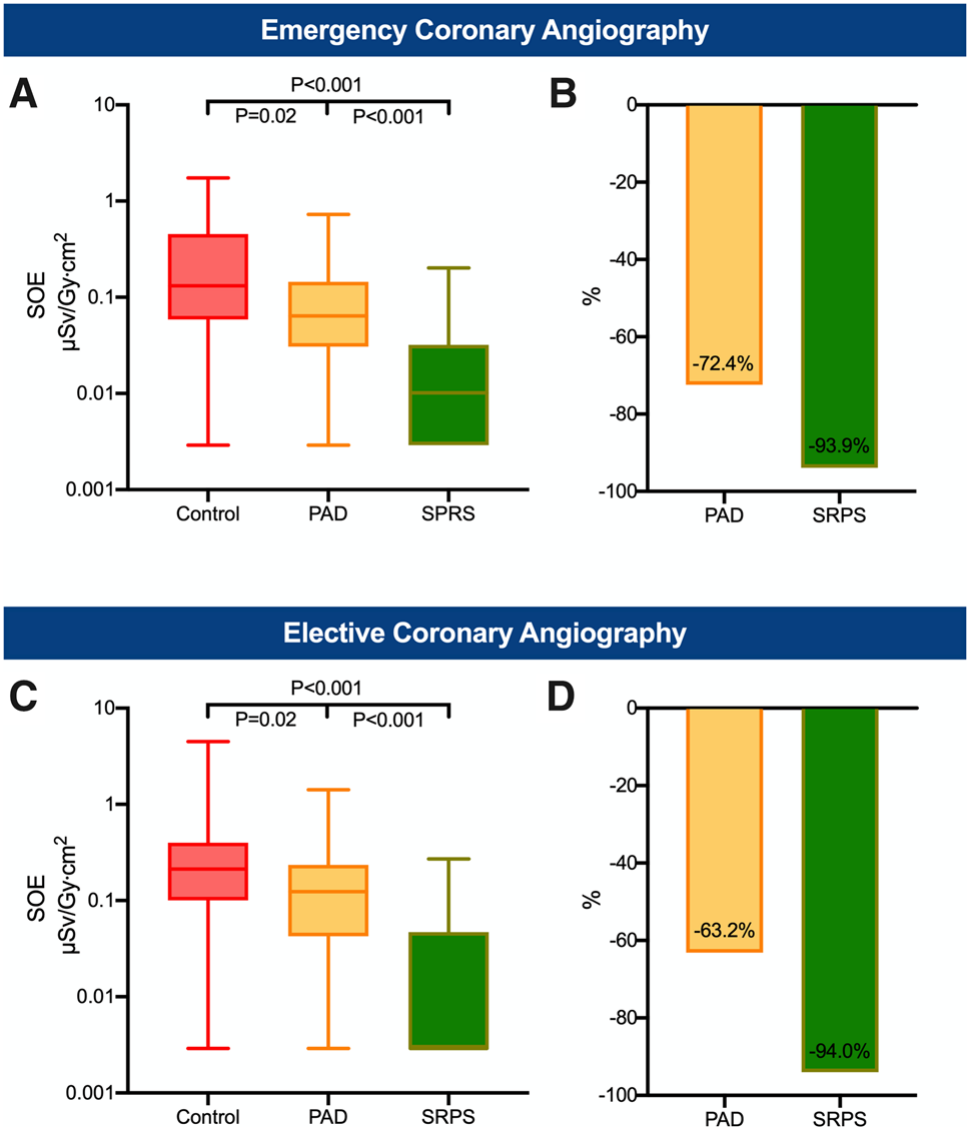
Emergency and elective procedures. Overall (head and chest) standardized operator exposure (SOE) received by the first operator during emergency (**A**) and elective procedures (**C**). The lower whisker shows the minimum and the upper whisker the maximum. Boxes extend from the 25th to the 75th percentile. Horizontal line in the box shows the median (50th percentile). Values with SOE = 0 µSv/Gy cm^2^ were replaced with half of the minimum detectable SOE value for illustration on the log-scale. Bar charts show the overall relative reduction in the mean SOE (**B**) during emergency procedures as well as during elective procedures (**D**). *PAD* protective scatter-radiation absorbing drapes, *SRPS* suspended radiation protection system

**Fig. 5:**
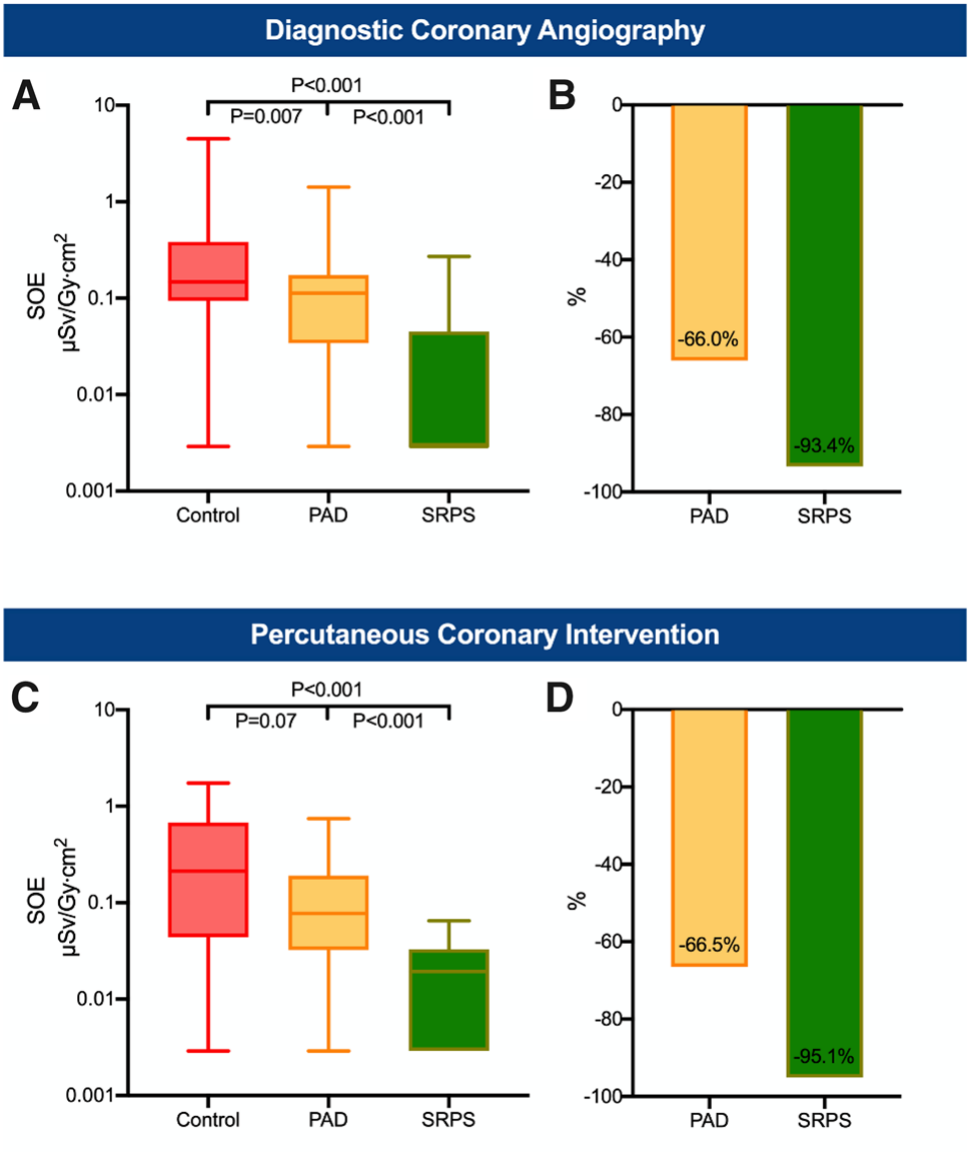
Diagnostic coronary angiography and percutaneous coronary intervention. Overall (head and chest) standardized operator exposure (SOE) received by the first operator during diagnostic procedures (**A**) and procedures with stenting (**C**). The lower whisker shows the minimum and the upper whisker the maximum. Boxes extend from the 25th to the 75th percentile. Horizontal line in the box shows the median (50th percentile). Values with SOE = 0 µSv/Gy cm^2^ were replaced with half of the minimum detectable SOE value for illustration on the log-scale. Bar charts show the overall relative reduction in the mean SOE during diagnostic procedures (**B**) as well as during procedures with PCI (**D**). *PAD* protective scatter-radiation absorbing drapes, *SRPS* suspended radiation protection system

**Fig. 6:**
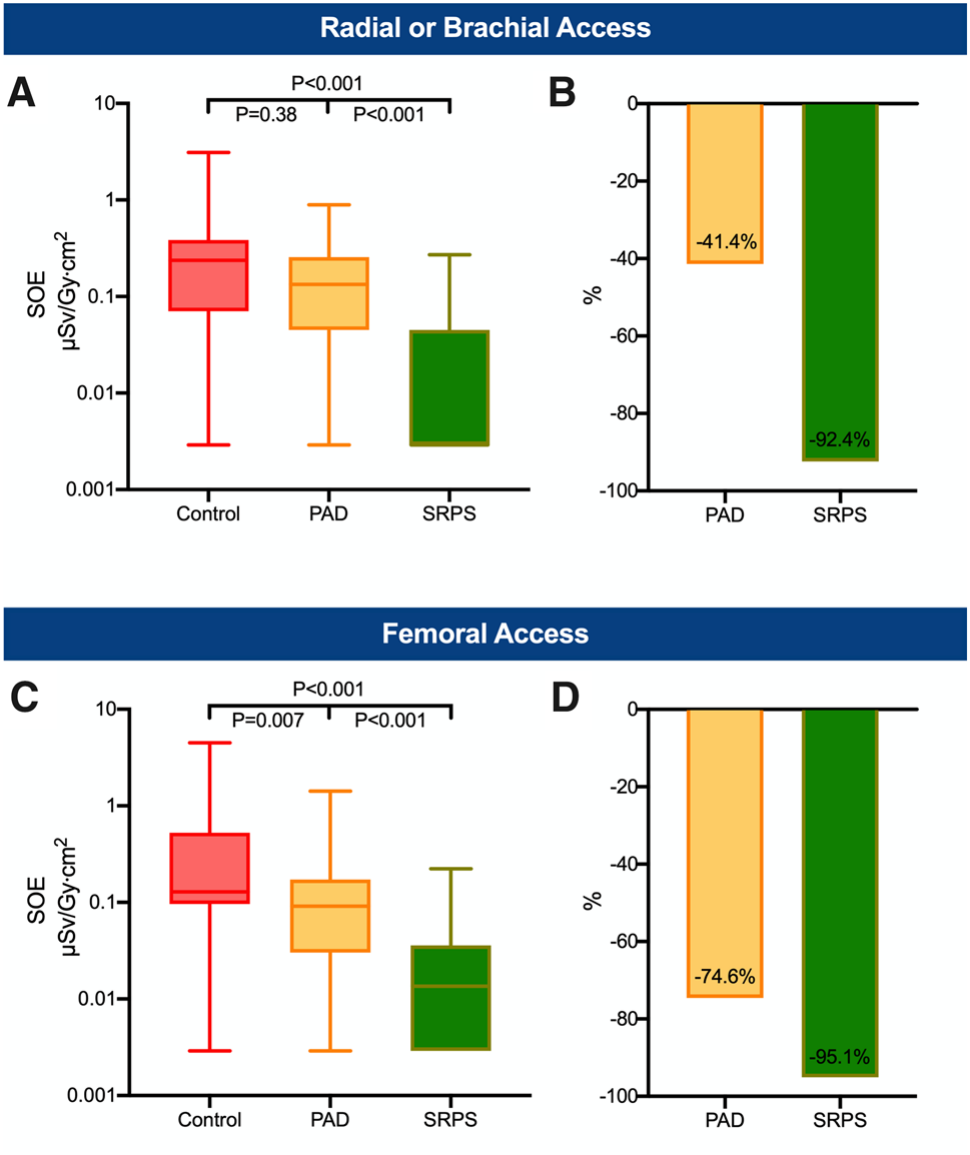
Radial/brachial and femoral access. Overall (head and chest) standardized operator exposure (SOE) received by the first operator in procedures with radial/brachial (**A**) and femoral access (**C**). The lower whisker shows the minimum and the upper whisker the maximum. Boxes extend from the 25th to the 75th percentile. Horizontal line in the box shows the median (50th percentile). Values with SOE = 0 µSv/Gy cm^2^ were replaced with half of the minimum detectable SOE value for illustration on the log-scale. Bar charts show the overall relative reduction in the mean SOE (**B**) during procedures with radial/brachial access as well as the during procedures with femoral access (**D**). *PAD* protective scatter-radiation absorbing drapes, *SRPS* suspended radiation protection system

Categorical variables are given as frequency and percentage. Continuous variables are given as median with interquartile range. Shapiro–Wilk test was used to test for distribution of normality. For multiple group comparisons the Kruskal–Wallis test was executed. All statistical tests were two-sided and a *P* < 0.05 was considered statistically significant. The SOE (Figs. 2, 3, 4, 5, 6) was compared with the Mann–Whitney *U* test with Bonferroni correction for multiple testing (*P* value threshold set at *P* < 0.017 accounting for 3 independent comparisons). Statistical analyses were performed with SPSS (version 28.0, IBM, Armonk, NY, USA) and figures were created with Prism (version 8, GraphPad Software, LLC, USA).

## Results

### Patients’ and procedural characteristics

A total of 229 patients undergoing CAG were assigned either to the control group (*N* = 74), PAD group (*N* = 82) or SRPS group (*N* = 73). There were no significant differences regarding BMI, median fluoroscopy time and DAP between the control, PAD and SRPS groups. Overall, PCI was performed in 66 cases and 145 had femoral access. Patients’ and procedural characteristics are summarized in Table 1.

**Table 1:** Patients' and procedural characteristics

Characteristic	Control	PAD	SRPS	*P* value
	*N* = 74	*N* = 82	*N* = 73	
DAP—Gy cm^2^	22.03 (12.07–36.18)	31.70 (15.83–58.86)	22.14 (13.84–42.75)	0.07
Fluoroscopy time—min	6.00 (4.01–11.44)	9.94 (4.39–14.22)	7.67 (4.38–13.34)	0.18
BMI—kg/m^2^	27.14 (23.71–30.40)	26.71 (23.88–29.15)	27.50 (25.05–29.55)	0.40
Cardiovascular risk factors—no. (%)				
Arterial hypertension	41 (55.4)	49 (59.8)	48 (65.8)	0.44
Diabetes mellitus	20 (27.0)	36 (43.9)	26 (35.6)	0.09
Dyslipidemia	46 (62.2)	47 (57.3)	47 (64.4)	0.65
Smoking^a^	35 (47.3)	32 (39.0)	34 (46.6)	0.51
Family history of CAD	16 (21.6)	20 (24.4)	23 (31.5)	0.37
Setting of CAG—no. (%)				
Emergency CAG	25 (33.8)	31 (37.8)	24 (32.9)	0.79
Elective CAG	49 (66.2)	51 (62.2)	49 (67.1)	0.79
Procedural characteristics—no. (%)				
Radial or brachial access	40 (54.1)	13 (15.9)	31 (42.5)	< 0.001
Femoral access	34 (45.9)	69 (84.1)	42 (57.5)	< 0.001
PCI	17 (23.0)	27 (32.9)	22 (30.1)	0.37
Lesions treated with PCI—no. (%)				
Single-vessel disease	15 (88.2)	23 (85.2)	19 (86.4)	0.96
Multi-vessel disease	2 (11.8)	4 (14.8)	3 (13.6)	0.96
LMCA	1 (5.9)	3 (11.1)	0 (0.0)	0.27
LAD	11 (64.7)	17 (63.0)	9 (40.9)	0.21
LCX	2 (11.8)	5 (18.5)	6 (27.3)	0.47
RCA	6 (35.3)	8 (29.6)	10 (45.5)	0.52

*BMI* body mass index, *CAD* coronary artery disease, *CAG* coronary angiography, *DAP* dose area product, *LAD* left anterior descending artery, *LCX* left circumflex artery, *LMCA* left main coronary artery, *PAD* protective scatter-radiation absorbing drapes, *PCI* percutaneous coronary intervention, *RCA* right coronary artery, *SRPS* suspended radiation protection system

^a^Current smoker or former smoker

### Primary endpoint: SOE of the first operator

Compared with the control group, the overall SOE (Fig. 2A, B) was reduced by 93.9% in the SRPS group (*P* < 0.001) and by 66.4% in the PAD group (*P* < 0.001). Moreover, a relative reduction in the mean SOE of 81.9% (*P* < 0.001) between the SRPS and the PAD group was observed. At eye-level, the SOE (Fig. 2C, D) was significantly reduced in the SRPS group (87.7%, *P* < 0.001) and the PAD (40.6%, *P* < 0.001) group when compared to the control group. Furthermore, there was a significant reduction of 79.3% for the eye-level data between the SRPS and PAD group (*P* < 0.001). At chest level, there was a reduction of 96.9% in the SRPS (*P* < 0.001) and a 78.6% (*P* = 0.12) reduction in the PAD group (Fig. 2E, F). Supplemental Table 1 shows the 25%-quartile, the median and the 75%-quartile of all groups.

### Secondary endpoint: SOE of the assistant

The overall SOE values for the assistant (*N* = 125) were 38.0% lower in the SRPS (*P* = 0.83) and 30.6% lower in the PAD group (*P* = 0.98) than in the control group (Fig. 3A, B). Individual exposure data for the assistant at eye-level are shown in Fig. 3C–D and in Fig. 3E–F for the chest level.

### Subgroup analyses

A substantial reduction in the SOE was observed for emergency and elective procedures (Fig. 4). In emergency procedures, the overall SOE of the first operator was decreased by 93.9% in the SRPS group (*P* < 0.001, Fig. 4A, B) and by 72.4% (*P* = 0.02, Fig. 4A, B) in the PAD group compared to the control group. Similar findings were observed in elective procedures in which the overall SOE was reduced by 94.0% in the SRPS group (*P* < 0.001, Fig. 4C, D) and by 63.2% in the PAD group (*P* = 0.02, Fig. 4C, D). For diagnostic angiographies without subsequent intervention, the overall SOE was 93.4% lower in the SRPS group (*P* < 0.001, Fig. 5A, B) and 66.0% lower in the PAD group (*P* = 0.007, Fig. 5A, B) compared to the control group. In cases with coronary intervention, substantial reduction of 95.1% in the SRPS group (*P* < 0.001) and 66.5% in the PAD group (*P* = 0.07) was observed (Fig. 5C, D). Lastly, vascular access from different sites was analyzed (Fig. 6). In radial/brachial procedures the overall SOE was 92.4% lower in the SRPS group (*P* < 0.001, Fig. 6A, B) and 41.4% in the PAD group (*P* = 0.38, Fig. 6A, B). Similarly, in femoral procedures the overall SOE was 95.1% lower in the SRPS group (*P* < 0.001, Fig. 6C, D) and 74.6% lower in the PAD group (*P* = 0.007, Fig. 6C, D) than in the control group.

### Cost analysis

Compared to the standard protection, both PAD and the SRPS are associated with additional costs. The comparison between PAD and SRPS is based on the conditions of the Swiss health care system. Since the PAD is a disposable, every use is associated with the cost of a single unit (approx. 47 CHF). The SRPS requires an investment for acquisition and installation (approx. 85,000 CHF) and additional cost for the sterile cover for every use (approx. 32 CHF). Since the sterile cover is cheaper than a PAD, the initial investment of the SRPS is amortized after approximately 5600 procedures. For every additional 1000 procedures, the SRPS reduces the costs for radiation protection by approx. 15,000 CHF compared to the PAD.

## Discussion

This real-world study including all-comer procedures performed in the cardiac catheterization laboratory demonstrates that both the SRPS and the PAD provide improved protection from scatter radiation when compared with conventional radiation protection measures. In direct comparison, the SRPS provided significantly higher protection with 94% overall SOE reduction of scatter radiation while the PAD achieved 66% reduction. The effect was similar in diagnostic and interventional procedures as well as in elective and emergency interventions.

The well-studied effects of radiation on overall malignancy occurrence and the clustered occurrence of left sided brain tumors in case-series of interventional cardiologists suggest a causal relationship between occupational radiation exposure and the occurrence of malignancies [7, 8, 13]. Therefore, reduction of scatter radiation at the level of the head is of particular importance. While radiation protection glasses are an effective way to reduce the risk of cataracts, cranial radioprotective surgical caps designed to specifically reducing brain exposure showed no impact on brain dose distribution [14, 15] because of upward scatter radiation that enters the skull through the neck. Therefore, there is a clinical need for improved radiation protection for the head. Interestingly, the discrepancies between the protective effects of SRPS and the PAD are most prominent at the head level with 87.7% SOE reduction in the SRPS and only 40.6% reduction in the PAD group. These results imply that suspended radiation protection systems provide better protection from adverse effects that scatter radiation has on the head and the brain.

A clinical feature of the procedure that influenced the effectiveness of radiation protection by the PAD was the choice of the vascular access site. While the PAD achieved 74.6% overall SOE reduction for femoral access, only 41.4% reduction where documented when a radial access was chosen. The site dependence of the protection provided by protective drapes has already been shown in a previous study by Sciahbasi et al. [16] and represents a relevant shortcoming of this protection method. In contrast, the SRPS provides an overall SOE reduction of more than 92% independent of the vascular access site and cannot be displaced in complex and emergency cases requiring no attention to the placement and no repositioning during the intervention. Regarding the distribution of the vascular access sites, an over-representation of femoral access in the PAD group is apparent. Since this study did not control for the assignment of access sites to the groups, we can only hypothesize about potential reasons for this imbalance, but operators’ prior knowledge about the beneficiary effect of the use of the PAD in the pelvis/groin region [16] may play a role. A femoral PAD, however, can be used independent of the vascular access, therefore, the over-representation of femoral PADs does not skew the results in favor of the PAD but approximate the optimal achievable protective effect of the PAD when always placed in the femoral region.

The secondary endpoint, the reduction of SOE for the assistant, was negative. While the numeric reduction of scatter radiation caused by PAD (30.6%) and SRPS (38.0%) was within the range of previous studies [17], statistically significance was not reached due to a large variability on the basis of overall low radiation exposure. While no direct comparison between the SRPS and the PAD has yet been performed, previous studies demonstrated substantial protective effects for each of the protective devices during different types of interventional procedures [9, 10, 18–20]. Vlastra et al. demonstrated a relative SOE reduction of 20% by the PAD [10], which is substantially lower than the 66% SOE reduction observed herein. The substantial differences may be caused by differences in dosimeter placements, which were worn outside the lead apron in the study by Vlastra et al. [10]. A study by Ray et al. on the protective effects of SRPS at eye level showed results which are comparable with our observations. The authors reported a relative reduction in SOE of 99% compared with the use of table shields for procedures in interventional radiology [9]. However, cardiac catheterization involves deviating radiation exposure and may demand alternative settings and positions on the catheterization table compared to radiological interventions.

Besides the reduction of scatter radiation, the additional effort for the cathlab team and limitations for the operators are important clinical features of the radiation protection equipment that are hard to quantify but still important to discuss. Compared to a PAD which requires only a few seconds for correct placement the SRPS requires additional procedural efforts. In particular the placement of the sterile cover over the SRPS is an additional time-consuming step. In our experience, however, trained staff can mount the cover in 90–120 s and the step can be performed prior to the patient’s arrival in the cathlab so that no time is lost if urgent revascularization is needed. Depending on the type of suspension, there is only little to no impairment of freedom of movement for the operator at the table. In case the operator needs to leave the table, he/she can easily dis- and reconnect to the SRPS by a magnetic lock.

Despite the initial investment for acquisition and installation, the SRPS proved to be cost effective compared to the continuous use of PAD after 5600 procedures in the Swiss health care system. While the numbers may be different in other health care systems, the long-term economic advantage of the SRPS persists as long as the costs per use are lower than the cost of a PAD. The additional cost of less days lost from work due to orthopedic illnesses cannot be quantified with this study but may be significant given the high prevalence of lower back pain in interventional cardiologists.

Limitations of the study are the single center design with direct assignment in a 1:1:1 fashion instead of randomization for saving of time, in particular in emergency situations. However, standardization to DAP adjusts for potential confounders due to patients’ and procedural characteristics.

## Conclusion

In conclusion, this first head-to-head comparison showed that both the SRPS and the PAD enhance radiation protection significantly compared to conventional protection. In most clinical scenarios, the protective effect of SRPS is significantly higher than the additional protection provided by the PAD. Due to the non-randomized nature of the study, the results need to be seen as hypothesis-generating and further randomized, multicenter trials are warranted.

## Supplementary Information

Below is the link to the electronic supplementary material.

Supplementary file1 (DOCX 2866 kb)

## Data Availability

The datasets used and analysed during the current study are available from the corresponding author on reasonable request.
