# Supplementary material for: Effectiveness of radiation protection systems in the cardiac catheterization laboratory: a comparative study
Source: Clin Res Cardiol. 2023 Jan 16;112(5):605–13. doi: 10.1007/s00392-022-02142-8 (PMC10160176; doi:10.1007/s00392-022-02142-8)
Supplement: Supplementary file 1 — Supplementary file1 (DOCX 2866 kb) [file 392_2022_2142_MOESM1_ESM.docx]

**Supplementary Figure 1.**


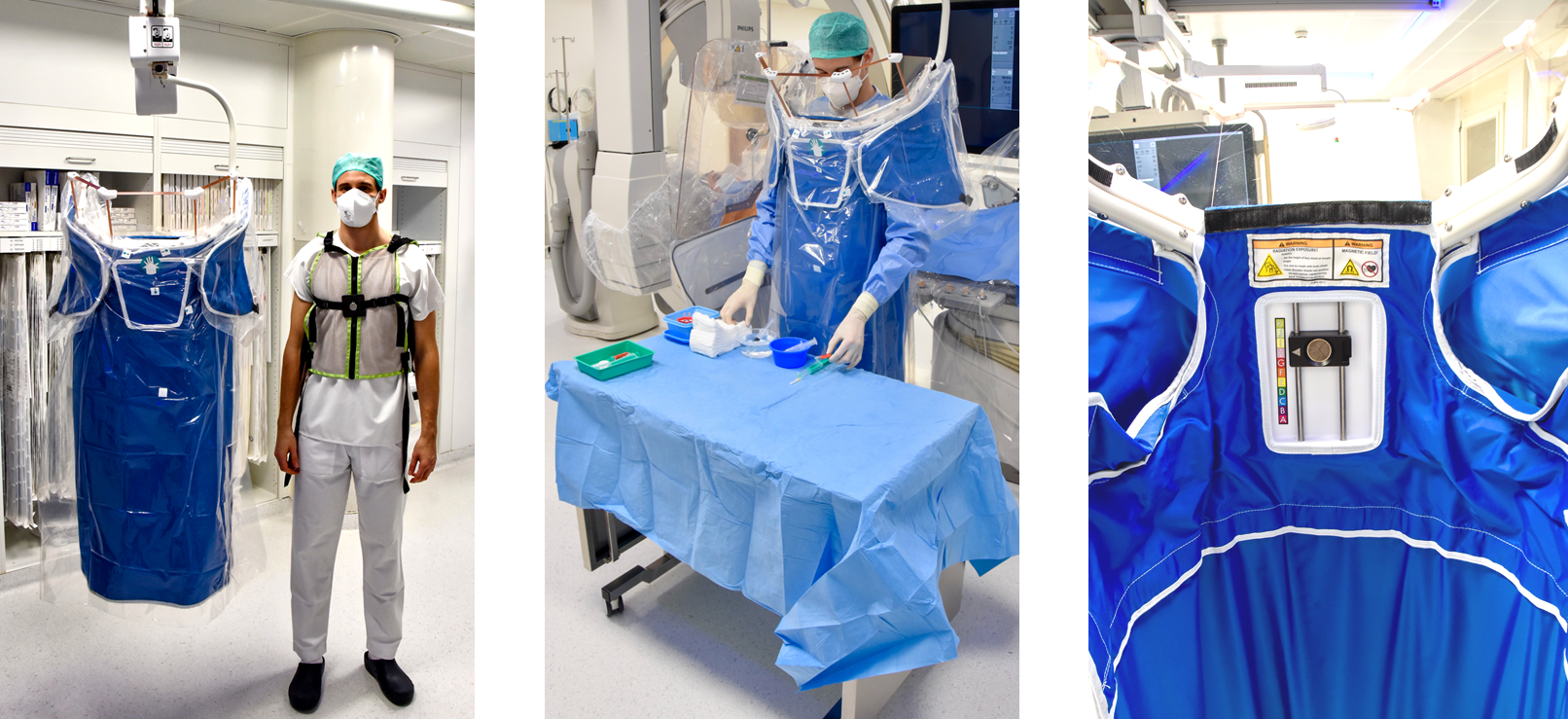


**Supplementary Table 1: Non-parametric representation of the standardized operator exposure for the first operator and the assistant**

|  | Control | | | PAD | | | SRPS | | |
| --- | --- | --- | --- | --- | --- | --- | --- | --- | --- |
| **SOE of the first operator** [µSv/Gy*cm^2^] | 25% quartile | median | 75% quartile | 25% quartile | median | 75% quartile | 25% quartile | median | 75% quartile |
| overall | 0.09 | 0.16 | 0.39 | 0.03 | 0.10 | 0.17 | 0.00* | 0.00* | 0.04 |
| head | 0.07 | 0.12 | 0.21 | 0.03 | 0.08 | 0.13 | 0.00* | 0.00* | 0.03 |
| chest | 0.00* | 0.00* | 0.18 | 0.00* | 0.00* | 0.05 | 0.00* | 0.00* | 0.00* |
| emergency procedures - overall | 0.07 | 0.13 | 0.38 | 0.03 | 0.06 | 0.13 | 0.00* | 0.01 | 0.03 |
| elective procedures - overall | 0.10 | 0.21 | 0.39 | 0.04 | 0.12 | 0.21 | 0.00* | 0.00* | 0.05 |
| diagnostic coronary angiographies - overall | 0.10 | 0.15 | 0.38 | 0.04 | 0.11 | 0.17 | 0.00* | 0.00* | 0.04 |
| procedures with PCI - overall | 0.05 | 0.21 | 0.57 | 0.03 | 0.08 | 0.17 | 0.00* | 0.02 | 0.03 |
| radial / brachial access - overall | 0.07 | 0.24 | 0.38 | 0.05 | 0.13 | 0.19 | 0.00* | 0.00* | 0.04 |
| femoral access - overall | 0.10 | 0.13 | 0.47 | 0.03 | 0.09 | 0.17 | 0.00* | 0.01 | 0.03 |
| **SOE of the assistant** [µSv/Gy*cm2] |  |  |  |  |  |  |  |  |  |
| overall | 0.05 | 0.11 | 0.22 | 0.06 | 0.13 | 0.17 | 0.07 | 0.11 | 0.17 |
| head | 0.03 | 0.06 | 0.12 | 0.03 | 0.05 | 0.08 | 0.04 | 0.06 | 0.11 |
| chest | 0.00* | 0.00* | 0.08 | 0.00* | 0.04 | 0.11 | 0.00* | 0.04 | 0.07 |

PAD protective scatter-radiation absorbing drapes; PCI percutaneous coronary intervention; SOE standardized operator exposure; SRPS suspended radiation protection system. *Radiation dose below the detection limit of the real time dosimeter.
